# Correlation between PTSD and sleep quality in community-dwelling elderly adults in Hunan province of China

**DOI:** 10.3389/fpsyt.2022.978660

**Published:** 2022-10-17

**Authors:** Jiri Cao, Yang Zhou, Man-Man Su, Wen-Hui Chen

**Affiliations:** ^1^Xiangya Medical School of Central South University, Changsha, China; ^2^Teaching and Research Section of Clinical Nursing, Xiangya Hospital of Central South University, Changsha, China; ^3^Department of Nursing, Xiangya Hospital of Central South University, Changsha, China; ^4^Department of Operating Room, Xiangya Hospital of Central South University, Changsha, China

**Keywords:** post-traumatic stress disorder (PTSD), sleep disorders, psychology, Post-Traumatic Growth (PTG), elderly adults

## Abstract

**Background:**

To understand the occurrence of post-traumatic stress disorder (PTSD) and the current status of sleep quality among community-dwelling elderly adults in Hunan Province of China, to explore the correlation between the two, and to analyze the trend of sleep disorders in PTSD elderly adults.

**Methods:**

A simple random sample containing 1,173 community-dwelling elderly adults in Hunan Province was established between March and May 2022, and an on-site face-to-face survey was administered using the PTSD Checklist–Civilian Version (PCL-C) with good reliability and validity, the Pittsburgh Sleep Quality Index (PSQI) scale, and a self-designed general condition questionnaire.

**Results:**

The incidence of PTSD in the 1,173 participants was 14.3% (168/1,173). The total incidence of sleep disorders was 40.9% (480/1,173); more specifically, the incidence of sleep disorders in participants with no PTSD symptom, in participants with mild-to-moderate PTSD symptoms, and in participants with severe PTSD symptoms was 36.3, 69.8, and 66.7%, respectively. The Spearman's rank correlation analysis showed that the total PTSD score and the scores of each dimension (i.e., re-experiencing symptom cluster, avoidance symptom cluster and hypervigilance symptom cluster) were positively correlated with the total PSQI score and its dimension scores (i.e., sleep quality, time to fall asleep, sleep duration, sleep efficiency, sleep disturbance, hypnotic medication, and daytime function) (*P* < 0.05). The correlation coefficients ranged from 0.013 to 0.495. For all PSQI dimensions, the differences across participants with different degrees of PTSD were statistically significant (*P* < 0.05).

**Conclusions:**

The overall status of PTSD and sleep quality in community-dwelling elderly adults in Hunan Province was not optimistic. The elderly with PTSD were more prone to sleep disorders, and the more severe the symptoms of PTSD, the poorer the sleep quality was. However, differences were observed in the scores of each dimension of sleep across participants with different degrees of PTSD. Regardless of the degree of PTSD symptoms, the sleep quality of the elderly is severely affected, and the occurrence rate is not unlimited.

## Introduction

Post-traumatic stress disorder (PTSD) refers to a class of mental disorders with delayed onset and persistent existence that occur after an individual experiencing, witnessing, or encountering one or more actual deaths, or threat of death, or serious injury, or threat to bodily integrity involving oneself or others. PTSD is now the fourth most common mental disorder in human beings (1). The prevalence of PTSD differs between different countries and between civilian and military population (2). However, the lifetime prevalence of PTSD in the general population is estimated at about 8% (3). The prevalence of combat-related PTSD in the United States is ~11–20% (4). Sleep disturbances are considered as a core feature of PTSD (5). It is noteworthy that sleep disorder commonly occurs after PTSD, while long-term suffering of sleep disorder may seriously affect the biological rhythm of the human body and even lead to symptoms such as endocrine disorders, gastrointestinal disorders, neurasthenia, and decreased immune function (6). To date, there are few studies on the relationship between PTSD and sleep quality targeting at the community-dwelling elderly adults. Therefore, the aims of this study were twofold: (a) to assess the overall level of sleep quality and PTSD among Community-dwelling elderly adults and (b) to explore the correlation between the two.

## Materials and methods

### Study design, study area, and period

A community-based cross-sectional study design was employed from March and May 2022. The study was conducted in three communities named “Tianxin,” “Xiangtan,” and “Yueyang” community, which are located in Hunan Province of China.

### Source population

These places were chosen because they were representative of communities in Hunan Province. All elderly adults were living in “Tianxin,” “Xiangtan,” and “Yueyang” community. They had lived in the communities for at least 6 months.

### Study population

Those who were available during the data collection period.

### Inclusion and exclusion criteria

Elderly adults from 3 communities in Hunan Province were simple randomly selected between March and May 2022 as the research objects. The inclusion criteria were as follows: (1) Age ≥ 65 years; (2) Community residents (>6 months) in Hunan Province of China; (3) Normal communication skills; (4) Participation on a voluntary basis.

The exclusion criteria were: (1) Patients with severe organic mental disorders or undergoing psychiatric treatment; (2) Patients with cognitive impairment.

### Sample size determination

The minimum sample size was calculated by the equation of descriptive analysis sample size estimation $n={\left(\frac{u_{a/2}\sigma }{\delta }\right)}^{2}$, which was equal to 722. Considering a dropout rate of 20%, the target sample size was 867. Eventually, 1,173 elderly individuals who met the inclusion criteria were included.

### Sampling procedure

**Figure 1:**
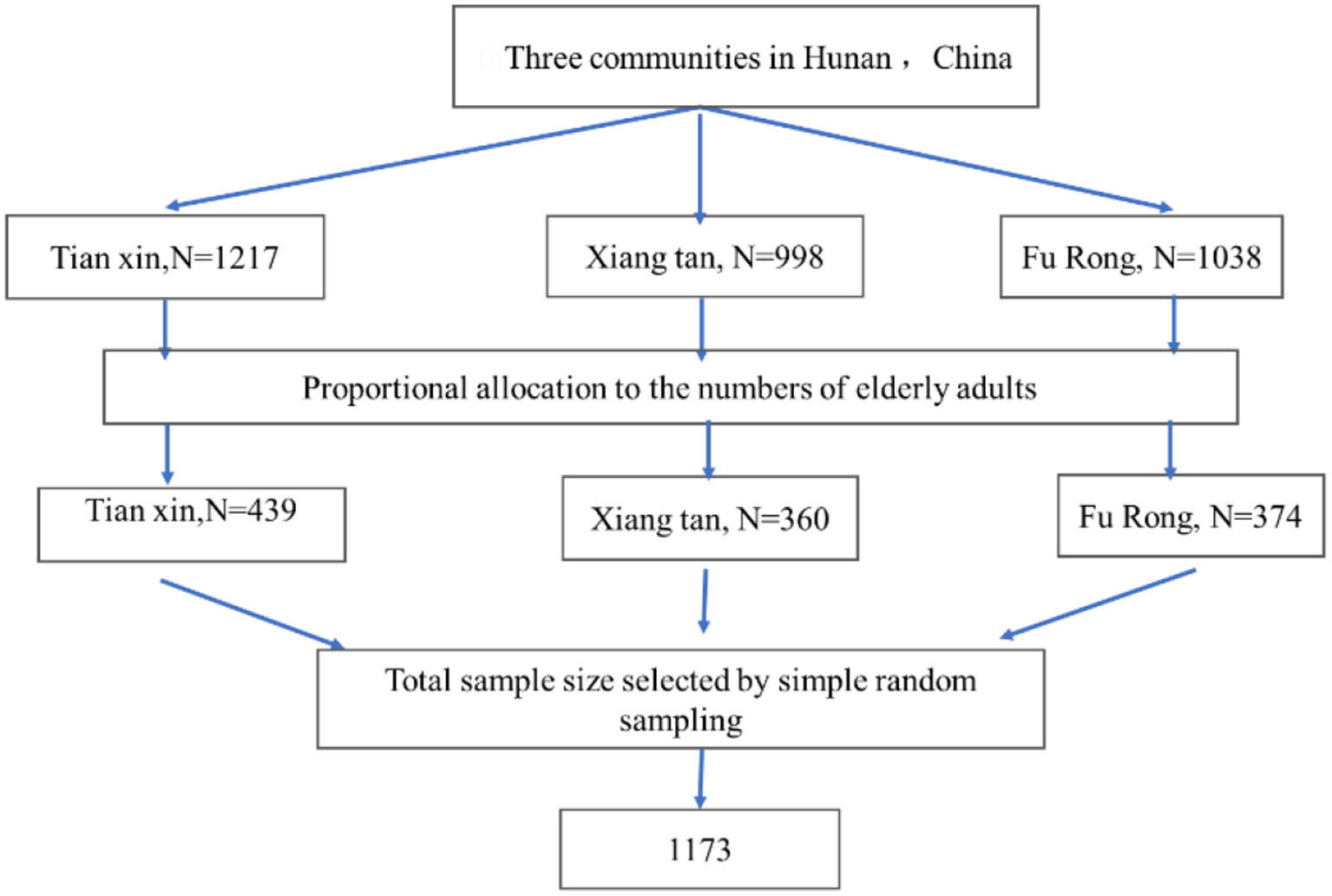
The flowchart of this study.

The sample of the study was drawn from the three communities in Hunan Province of China. The total calculated sample size (1,173 elderly adults) was proportionally allocated to each community based on the number of their elderly adults. A computer-generated simple random sampling method was employed to select elderly adults by using their sampling frame from each community (Figure 1).

### Methods

#### Survey tools

##### PTSD checklist–civilian version (PCL-C)

The PCL-C checklist was compiled by the Behavioral Sciences Branch of the American PTSD Research Center according to the fourth edition of the Diagnostic and Statistical Manual of Mental Disorders (7). It contains 17 items in total, corresponding to the 17 diagnostic criteria of PTSD, including three symptom clusters, namely the re-experiencing symptom cluster, the avoidance symptom cluster, and the hypervigilance symptom cluster. A five-point (1–5) scoring system is adopted, which means the cumulative total score ranges from 17 to 85 points (the higher the score, the more obvious the PTSD symptoms are). Many Chinese scholars agree to set 38 points as the critical standard (8). In this study, we also chose 38 points as the positive screening threshold, and the specific classification is as follows: 17–37 points indicating no symptom; 38–49 points indicating mild-to-moderate symptoms; ≥50 points indicating severe symptoms, which may be diagnosed as PTSD (9).

##### Pittsburgh sleep quality index (PSQI)

The PSQI scale was compiled by Buysse et al. of the University of Pittsburgh in 1989, which is mainly used to measure the sleep quality of an object in the recent 1 month (10). It contains 24 items in total, including 19 self-assessment items and 5 other-assessment items; 18 out of the 19 self-assessment items constitute 7 dimensions, namely subjective sleep quality, time to fall asleep, sleep duration, sleep efficiency, sleep disturbance, use of hypnotic medication, and daytime dysfunction. Each dimension is scored from 0 to 3, making the total score ranging from 0 to 21 points (the higher the score, the poorer the sleep quality is). The Chinese version of PSQI that is currently used in China was localized by Liu and Tang (11). At present, most studies set a score >7 as the critical value, i.e., the diagnostic standard for sleep disorder. The Cronbach's coefficient of this scale is 0.758.

#### Data collection

In general, the questionnaire survey method was adopted in this study. Members of the research team who had accepted unified training visited different communities. The training was provided to each local community data collector for 1 day on assessment tools, how to collect data using the tools, methodology, ethical concerns, and how to supervise the data collection process. After obtaining the consent of the participants, the questionnaire was answered under one-on-one guidance. It took about 20 min to complete. The questionnaires were collected upon completion onsite. A pretest was done before the actual study on 5% (45) of the participants to identify potential problems in data collection tools and modification of the questionnaire. Data collectors were supervised, and the filled questionnaires were checked daily. Each day throughout the data collection period, the completed questionnaires were assured of completeness and consistency. The collected data were entered into the computer, then checked and processed appropriately. Initially, 1,200 elderly adults consented to participate in the study and received the questionnaires. Of them, 1,173 elderly adults completed the questionnaire, resulting in a response rate of 97.8%. This study had been reviewed and approved by the ethics committee of the research unit, and the participant's privacy and data security were properly guaranteed.

### Statistics method

All the data was processed and analyzed using SPSS 26.0. The original data was checked and entered into the software by two members of the research team, and then was further verified by the two members. The statistical description of measurement data was expressed as ($\bar{X}\pm S$). The PCL-C total score and dimension scores were compared between the sleep disorder group and the no sleep disorder group by the Mann-Whitney *U*-test with two independent samples. The PSQI total score and dimension scores were compared among the three groups representing different degrees of PTSD symptoms by the Kruskal-Wallis H test with multiple samples. The statistical description of enumeration data was expressed by frequency and percentage. The percentage comparison between two or more groups was implemented by χ^2^ test. The Spearman correlation analysis was used to analyze the relationships between the PTSD and PSQI scores and their scores in each dimension. *P* < 0.05 indicated a statistically significant difference.

## Results

The participants' demographic characteristics are shown in Table 1.

**Table 1:** Demographics of the participants (*n* = 1,173).

**Variables**	**Mean ± SD**	***n*%**
**Age**	**72.74** **± 6.47**	
65~74		800 (68.2)
75~89		351 (29.9)
90 and above		22 (1.9)
**Gender**		
Female		629 (53.6)
Male		544 (46.4)
**Body Max Index (BMI)**		
Under weight (<18.5)		101 (8.6)
Normal (18.5~23.9)		666 (56.7)
Overweight (24~26.9)		288 (24.6)
Obese (≧27)		118 (10.1)
**Educational level**		
No formal education or primary education		533 (45.4)
Secondary education		526 (44.8)
Tertiary education		1 14 (9.7)
**Marital status**		
Single		15 (1.3)
Married		896 (76.4)
Divorced		15 (1.3)
Widows		247 (21.1)
**Fertility status**		
Childless		28 (2.4)
One child		214 (18.2)
Two children		489 (41.7)
3 children and above		442 (37.7)
**Pre-retirement occupation**		
Unemployed		240 (20.5)
Mental labor		342 (29.2)
Physical labor		591 (50.4)
**Monthly personal income (RMB)**		
<1,000		304 (25.9)
1,000~3,000		1000~3000
3,000~5,000		290 (24.7)
>5,000		92 (7.8)
**Religion**		
No		1 108 (94.5)
Yes		65 (5.5)
**Smoking**		
Smoker		265 (22.6)
Non-smoker		908 (77.4)
**Physical activity level**		
No exercise		280 (23.9)
1~2 times per week		371 (3 1.6)
≧3 times per week		137 (1 1.7)
Exercise almost every day		385 (32.8)

### Overall status of PCL-C and sleep quality PSQI in community-dwelling elderly adults with varying degrees of PTSD

#### Incidence of PTSD in community-dwelling elderly adults

In this study, the mean PCL-C total score of PTSD was (26.78 ± 10.96), and the incidence of PTSD symptoms was 14.3% (168/1,173). Among the 168 participants with PTSD symptoms, objects belonging to mild or moderate symptoms accounted for 57.1% (96/168), and those belonging to severe symptoms accounted for 42.9% (72/168). The PCL-C scores in each dimension were as follows: the mean score of re-experiencing symptom (7.86 ± 3.77), the mean score of avoidance symptom (10.59 ± 4.60), and the mean score of hypervigilance symptom (8.32 ± 3.49).

#### Incidence of PSQI in community-dwelling elderly adults

Among the study objects, the mean PSQI total score was (7.60 ± 5.14). Specifically, 480 participants had a PSQI total score >7 (with sleep disorders), accounting for 40.9% of the total. Scores of each PSQI factor were as follows: the mean score of subjective sleep quality (1.26 ± 0.77), the mean score of time to fall asleep (1.48 ± 1.02), the mean score of sleep duration (0.91 ± 1.08), the mean score of sleep efficiency (1.07 ± 1.22), the mean score of sleep disturbance (1.28 ± 0.62), the mean score of hypnotic medication (0.20 ± 0.66), and the mean score of daytime function (1.05 ± 0.97).

### Correlation analysis between PCL-C and PSQI

In this study, the PCL-C total score was positively correlated with the PSQI total score (*r* = 0.452, *P* < 0.001), that is, the more severe the PTSD symptoms in the elderly, the worse the sleep quality. The Spearman rank correlation analysis on the PCL-C and PSQI dimension scores showed that each PCL-C dimension (re-experiencing symptom, avoidance symptom, and hypervigilance symptom) was positively correlated with any of the PSQI dimensions (sleep quality, time to fall asleep, sleep duration, sleep efficiency, sleep disturbance, hypnotic medication, and daytime function) (*P* < 0.05 for all), with the correlation coefficients ranging from 0.013 to 0.495 (Table 2).

**Table 2:** Correlation analysis between PTSD and PSQI dimension scores (*n* = 1,173).

**Variable**	**Item**	**Sleep quality**	**Time to fall asleep**	**Sleep duration**	**Sleep efficiency**	**Sleep disturbance**	**Hypnotic medication**	**Daytime function**	**PSQI total score**
Re-experiencing symptom	*r_*s*_*	0.248	0.246	0.143	0.131	0.349	0.156	0.359	0.331
	*P*	0.000	0.000	0.000	0.000	0.000	0.000	0.000	0.000
Avoidance symptom	*r_*s*_*	0.236	0.228	0.109	0.080	0.352	0.136	0.415	0.308
	*P*	0.000	0.000	0.000	0.006	0.000	0.000	0.000	0.000
Hypervigilance symptom	*r_*s*_*	0.450	0.418	0.264	0.209	0.432	0.222	0.464	0.514
	*P*	0.000	0.000	0.000	0.000	0.000	0.000	0.000	0.000
PCL-C total score	*r_*s*_*	0.358	0.337	0.192	0.156	0.407	0.191	0.453	0.431
	*P*	0.000	0.000	0.000	0.000	0.000	0.000	0.000	0.000

### Sleep performance of elderly adults with varying degrees of PTSD

#### Comparison of the incidence of sleep disorders in elderly adults with varying degrees of PTSD

**Figure 2:**
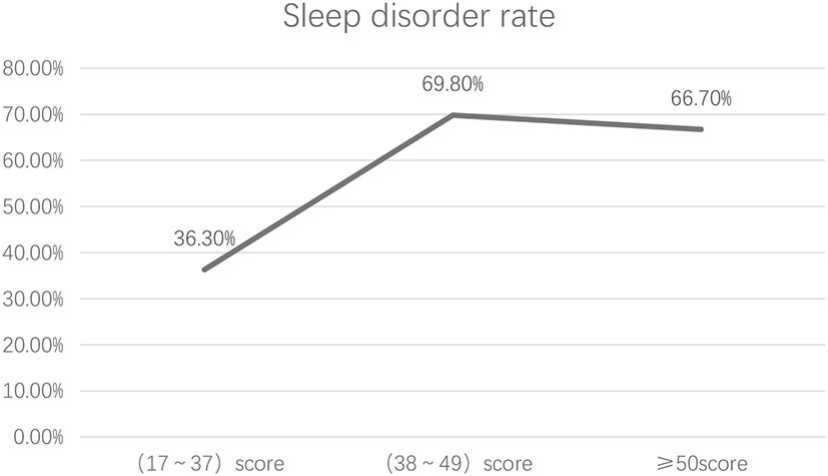
Developing trend of the incidence of PTSD sleep disorders in varying degrees.

Among the 1,173 community-dwelling elderly adults, 480 had sleep disorders, including 365 from those 1,005 participants without PTSD symptom; the overall incidence of sleep disorders was 36.3%. Among the 96 participants with mild or moderate PTSD symptoms, 67 had sleep disorders, and the incidence of sleep disorders was 69.8%. Among the 72 participants with severe PTSD symptoms, 48 had sleep disorders, and the incidence of sleep disorders was 66.7%. The overall developing curve showed an upward trend first and then entered a plateau (Figure 2). The incidence of sleep disorders corresponding to varying degrees of PTSD was tested by chi-square test, and the difference was statistically significant (χ2 = 61.645, *P* < 0.001) (Table 3).

**Table 3:** Comparison of the incidence of sleep disorders in elderly adults with varying degrees of PTSD (*n*%).

**Variable**	**Quantity**	**With sleep disorders**	**Without sleep disorder**	**χ^2^**	***P***
Without symptom	1,005	365 (36.3%)	640 (63.7%)	61.645	0.000
Mild and moderate symptoms	96	67 (69.8%)	29 (30.2%)		
Severe symptoms	72	48 (66.7%)	24 (33.3%)		

#### Comparison of the incidence of sleep disorders in elderly adults with and without PTSD symptoms (PCL-C score >38)

Among the 1,173 community-dwelling elderly adults, the incidence of sleep disorders was compared between elderly adults with and without PTSD symptoms. According to the chi-square test, the difference between the two groups was statistically significant (χ^2^ = 61.479, *P* < 0.001) (Table 4).

**Table 4:** Comparison of the incidence of sleep disorders between elderly adults with and without PTSD (*n*%).

**Variable**	**With sleep disorders**	**Without sleep disorder**	**χ^2^**	***P***
With PTSD symptoms	115	53	61.479	0.000
Without PTSD symptom	365	640		
Total	480	693		

#### Comparison of sleep PSQI dimensions in elderly adults with varying degrees of PTSD

The sleep status of elderly adults with varying degrees of PTSD was compared in terms of each PSQI dimension. The Kruskal-Wallis H test showed that there were statistically significant differences in all the seven PSQI dimensions between varying degrees of PTSD (*P* < 0.001) (Table 5).

**Table 5:** Comparison of sleep quality between elderly adults with varying degrees of PTSD in different dimensions (points, $\bar{X}\pm S$).

**Sleep factor**	**Without PTSD**	**Mild or moderate PTSD**	**Severe PTSD**	***H***	***P***
Subjective sleep quality	1.18 ± 0.75	1.63 ± 0.78	1.78 ± 0.75	67.860	0.000
Time to fall asleep	1.38 ± 1.02	2.06 ± 0.79	2.07 ± 0.89	66.860	0.000
Sleep duration	0.83 ± 1.04	1.38 ± 1.20	1.39 ± 1.19	36.538	0.000
Sleep efficiency	1.00 ± 1.20	1.53 ± 1.25	1.36 ± 1.32	20.948	0.000
Sleep disturbance	1.19 ± 0.57	1.79 ± 0.58	1.85 ± 0.76	137.637	0.000
Hypnotic medication	0.17 ± 0.63	0.27 ± 0.68	0.47 ± 0.90	26.420	0.000
Daytime function	0.92 ± 0.92	1.76 ± 0.97	1.86 ± 0.94	109.210	0.000

### Comparison of the PCL-C total score and each dimension score between elderly adults with and without sleep disorders

In this study, the mean PCL-C total score of community-dwelling elderly adults with sleep disorders was (30.75 ± 12.25), and that of those without sleep disorder was (24.02 ± 9.00). The PCL-C total score and each dimension score were compared between the participants with and without sleep disorders, and the Mann-whitney *U* test showed that those with sleep disorders had higher PCL-C total score, re-experiencing symptom score, avoidance symptom score and hypervigilance symptom score than those without sleep disorder, and the differences were statistically significant (*P* < 0.001) (Table 6).

**Table 6:** Comparison of PCL-C total score and each dimension score between elderly adults with and without sleep disorders ($\bar{X}\pm S$).

**Variable**	**With sleep disorders**	**Without sleep disorder**	***Z***	***P***
	***n* = 480**	**n = 693**		
Re-experiencing symptom	8.97 ± 4.28	7.10 ± 3.15	−7.957	0.000
Avoidance symptom	11.78 ± 5.17	9.77 ± 3.97	−7.285	0.000
Hypervigilance symptom	10.00 ± 3.86	7.16 ± 2.65	−13.826	0.000
PCL-C total score	30.7 ± 12.25	24.02 ± 9.00	−10.920	0.000

## Discussions

### The current status reflected by PTSD and PSQI was unoptimistic

The incidence of PTSD in this study (14.3%) was higher than that of the general population abroad. Surveys in other countries showed that the incidence of PTSD in the general population ranged from 1 to 14%, with an average of 8% (1). In China, the research on PTSD started relatively late, and the corresponding epidemiological investigations were limited. To date, the incidence of PTSD in the general population has not been reported yet, not to mention that in the community-dwelling elderly adults. The results of this study showed that the incidence of sleep disorders was 40.9%. The total PSQI score of the participants was (7.60 ± 5.14). This study was consistent with that reported by Dong et al. (45%) (12), which was much higher than the domestic norm (13). The results of this study indicated that the sleep quality among community-dwelling elderly adults was generally low. Sleep disorders of varying degrees were common and needed to be solved urgently. The incidence of PTSD in community elderly in China is slightly higher than that in foreign studies, and the impact of PTSD on sleep quality needs further study.

### Participants with PTSD symptoms had a high incidence of sleep disorders

Of the three major symptom clusters of PTSD, two are related to sleep: under hypervigilance symptoms, problems like difficulty to fall asleep, superficial sleep and sleep disruption are likely to occur; under re-experiencing symptoms, the repeated occurrence of traumatic scenes in dreams is also a symptom related to sleep disorder (14). Sleep problems were quite prominent in elderly adults with PTSD symptoms. Most people with PTSD would experience sleep disorders such as insomnia and excessive arousal (15). The results of this study showed that the incidence of sleep disorders among the community-dwelling elderly adults with PTSD symptoms was up to 68.4%, which was higher than that of the elderly without PTSD symptoms (36.3%). It implies that the community-dwelling elderly adults with PTSD symptoms are more prone to sleep disorders.

### Regardless of the degree of PTSD symptoms, the sleep quality of the elderly is severely affected, and the occurrence rate is not unlimited

The line chart of the incidence of sleep disorder in this study showed that the elderly without PTSD symptoms had a low incidence of sleep disorders, but as long as PTSD symptoms occurred, the incidence of sleep disorders would increase significantly. Severe PTSD was not consistently higher than moderate PTSD. This is because the occurrence of PTSD symptoms would disrupt the rapid eye movement sleep state and directly affect sleep quality (16). Meanwhile, some PTSD symptoms can be relieved naturally with the progress of time, or that Post-Traumatic Growth (PTG) occurs in the elderly with severe PTSD symptoms (17). PTG means that, after elderly adults suffered from stress and trauma (e.g., widowing, loss of child, surgery, disease, death of relatives and friends, etc.), they could receive care and support from family members, relatives, friends and society timely to help them recover. Moreover, the improvement of cognition and open-minded and optimistic characters could help them enhance their confidence in life and build positive personality (e.g., positive attitude toward life, promotion in mental health, etc.) (18). Therefore, the society should establish a good living environment for the elderly and pay more attention to them to increase their sense of support. At the same time, it is also necessary to provide the elderly with means of psychological catharsis and encourage them to actively reduce the occurrence of PTSD through mental consultation, allowing the elderly adults to return to the society with a positive attitude. This is helpful for improving their sleep quality.

### The PTSD and PSQI total scores and scores of each dimension were positively correlated

Sleep disorders of the community-dwelling elderly adults were closely related to the severity of PTSD symptoms (the correlation coefficient between the two was *r* = 0.452). Further analysis showed that the three dimensions of PTSD (i.e., re-experiencing symptoms, avoidance symptoms and hypervigilance symptoms) were significantly related to the PSQI score. In addition, they had a positive predictive effect on the PSQI score, but the predictive power differed among different dimensions. The overall trend indicated that the more severe the PTSD symptoms, the worse the sleep quality and sleep disorders were. This is consistent with the survey results of Yang et al. (8) on trauma patients in the Pearl River Delta region of China. However, differences were observed in the scores of each dimension of sleep among the elderly with different degrees of PTSD. The reason is that the re-experiencing symptoms, avoidance symptoms and hypervigilance symptoms would intensify the patients' degree of PTSD (19), which might eventually lead to symptoms like prolonged sleep latency, increased number of awakenings, shortened total sleep duration, and reduced fast-running sleep periods; consequently, the patients would suffer objective sleep disorder and their daytime function would be badly affected. Among the 7 dimensions of PSQI, the scores of sleep disturbance and daytime function were relatively higher, indicating that the elderly adults generally experienced poor sleep quality, which was not effectively controlled, resulting in sleepiness, fatigue, lack of energy the next day, and other problems that seriously affected their quality of life.

### Difficulty to fall asleep was the most serious problem across the elderly with all degrees of PTSD, and the use of sleep aids was not common

The scores of various dimensions of sleep quality across the elderly with different degrees of PTSD showed that, among the various dimensions of sleep quality, time to fall asleep had the highest score, suggesting that the difficulty to fall asleep was the most serious problem. This might be related to the combination of factors such as changes in sleep rhythm caused by body aging, longer bed rest time, and decreased daytime activity. On the other hand, the dimension of hypnotic medication had the lowest score, suggesting that the use of sleep aids was not common in the elderly.

### Interaction between PTSD and sleep

According to our results, the scores of the three-dimensions of PCL-C (i.e., re-experiencing symptoms, avoidance symptoms and hypervigilance symptoms) of the elderly with sleep disorders were significantly higher than those of the elderly without sleep disorders, which implies that the elderly with sleep disorders were more prone to PTSD. Sleep is an essential rhythmic physiological activity for the functioning of the human body. An earlier study showed that sleep could weaken the memory of traumatic events so as to minimize the corresponding symptoms (20). A good sleep can not only help maintain health condition and body energy, but also improve the body's immunity to facilitate physical recovery (21). On the contrary, sleep disorders are likely to result in lack of energy, aggravate physical and mental diseases, hinder the rehabilitation process, and seriously threaten the physical and mental health and life quality for the elderly. Li et al. (22) reported that sleep disorders were an important risk factor for PTSD. Insomnia and nightmares in sleep disorders have been consistently seen as manifestation of the re-experiencing and hypervigilance symptoms of PTSD. Therefore, improvement of the sleep disorder status is a feasible way to alleviate PTSD symptoms for elderly adults.

### Limitation of the study

The limitation of this study was that it was only a cross-sectional study and cannot determine the causal relationship. So it did not provide reference basis for improving the sleep quality of the elderly. Future research should conduct longitudinal follow-up studies. The use of a self-administered questionnaire that relied on subjective measures of sleep quality may have the possibility of recall bias.

## Conclusions

Elderly adults with PTSD were more prone to sleep disorders than the general elderly population, and the more severe the symptoms of PTSD, the worse the sleep quality was. However, among the elderly with different degrees of PTSD symptoms, the scores in various dimensions of sleep were different. Specifically, elderly adults with PTSD symptoms had a higher incidence of sleep disorders than those without symptoms. Regardless of the degree of PTSD symptoms, the sleep quality of the elderly is severely affected, and the occurrence rate is not unlimited. Overall, there were interactions and correlation between sleep quality and PTSD. Therefore, a higher importance should be attached to the physical and mental health and the sleep status of community-dwelling elderly adults. It will produce a positive effect on improving the life quality of the elderly, maintaining social stability, and promoting social harmony.

## Data availability statement

The original contributions presented in the study are included in the article/supplementary material, further inquiries can be directed to the corresponding author.

## Ethics statement

The studies involving human participants were reviewed and approved by the National Key Research and Development Program of China. The patients/participants provided their written informed consent to participate in this study.

## Author contributions

W-HC and YZ defined the focus of the review. W-HC and M-MS searched and screened the papers for inclusion. JC and W-HC summarized included papers, analysis the data, drafted, and revised the manuscript. All authors contributed to reviewing the manuscript, read, and approved the submitted version.

## Funding

This work was supported by the National Key Research and Development Program of China (No. 2020YFC2005300), Key R&D Project of Hunan Science and Technology (2020sk2075), and Research Project of Hunan Provincial Health Commission (202114050153 and 202214052636).

## Conflict of interest

The authors declare that the research was conducted in the absence of any commercial or financial relationships that could be construed as a potential conflict of interest.

## Publisher's note

All claims expressed in this article are solely those of the authors and do not necessarily represent those of their affiliated organizations, or those of the publisher, the editors and the reviewers. Any product that may be evaluated in this article, or claim that may be made by its manufacturer, is not guaranteed or endorsed by the publisher.
